# Soy-Based Soft Matrices for Encapsulation and Delivery of Hydrophilic Compounds

**DOI:** 10.3390/polym10060583

**Published:** 2018-05-26

**Authors:** Ruvimbo Chitemere, Shane Stafslien, Long Jiang, Dean Webster, Mohiuddin Quadir

**Affiliations:** 1 Department of Coatings and Polymeric Materials, North Dakota State University, Fargo, ND 58108, USA; ruvimbo.chitemere@ndsu.edu; 2 Office for Research and Creative Activity, North Dakota State University, Fargo, ND 58108, USA; shane.stafslien@ndsu.edu; 3 Department of Mechanical Engineering, North Dakota State University, Fargo, ND 58108, USA; long.jiang@ndsu.edu

**Keywords:** drug delivery systems, controlled release, lipid matrices, epoxidized sucrose soyate

## Abstract

A new controlled-release platform for hydrophilic compounds has been developed, utilizing citric acid-cured epoxidized sucrose soyate (ESS) as the matrix forming material. By cross-linking epoxy groups of ESS with citric acid in the presence of a hydrophilic model molecule, sodium salt of fluorescein (Sod-FS), we were able to entrap the latter homogenously within the ESS matrix. No chemical change of the entrapped active agent was evident during the fabrication process. Hydrophobicity of the matrix was found to be the rate-limiting factor for sustaining the release of the hydrophilic model compound, while inclusion of release-modifiers such as poly(ethylene glycol) (PEG) within the matrix system modulated the rate and extent of guest release. Using 5 kDa PEG at 5% *w*/*w* of the total formulation, it was possible to extend the release of the active ingredient for more than a month. In addition, the amount of modifiers in formulations also influenced the mechanical properties of the matrices, including loss and storage modulus. Mechanism of active release from ESS matrices was also evaluated using established kinetic models. Formulations composed entirely of ESS showed a non-Fickian (anomalous) release behavior while Fickian (Case I) transport was the predominant mechanism of active release from ESS systems containing different amount of PEGs. The mean dissolution time (MDT) of the hydrophilic guest molecule from within the ESS matrix was found to be a function of the molecular weight and the amount of PEG included. At the molecular level, we observed no cellular toxicities associated with ESS up to a concentration level of 10 μM. We envision that such fully bio-based matrices can find applications in compounding point-of-care, extended-release formulations of highly water-soluble active agents.

## 1. Introduction

Extended-release formulations have been an attractive product platform for therapeutic and diagnostic applications [1]. Extending the release of water-soluble guest molecules is an unmet challenge in this area, since many therapeutically active drugs, diagnostic agents, biocides, and fungicides are formulated as the salt of the active molecule to impart maximum engagement with their substrate targets. A set of existing technologies for fabricating extended-release formulations for hydrophilic drugs and bioactive chemical moieties include direct-compression of the active compound into matrices using excipients, micro- and/or nano-encapsulation, or reversible chemical conjugation of the active species with a polymer. Among these fabrication processes, polymer-based matrix systems are commonly used for manufacturing extended- and controlled release delivery systems because it makes such manufacturing easy [2]. Using mechanically compressed or chemically cross-linked polymeric network to form a matrix, which is capable of suppressing the rate of diffusion of an entrapped molecule to the surrounding milieu, has been the basis of such controlled-release formulations.

**Figure 1:**
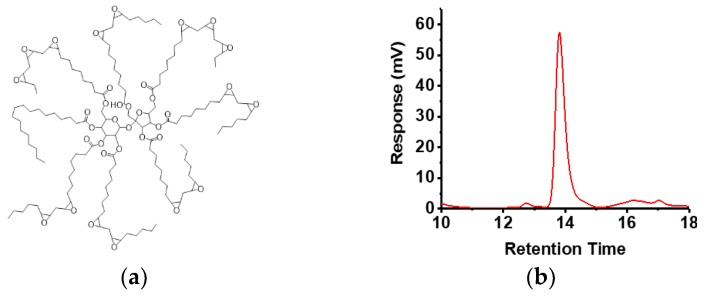

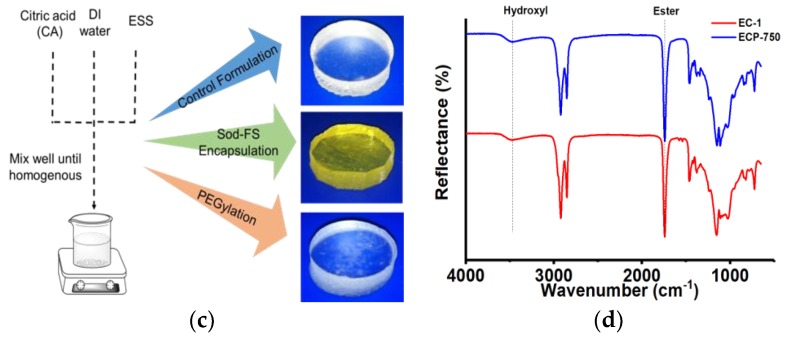
(**a**) Idealized structure of epoxidized sucrose soyate (ESS); (**b**) Gel permeation chromatograph (GPC) chromatogram of ESS; (**c**) general scheme for the preparation of crosslinked ESS matrices; (**d**) Attenuated Total Reflectance (ATR) spectra of EC-1 (control) matrix and ECP-750 (PEG modified) matrix.

Ideally, a matrix-forming polymer, intended for designing extended release formulations of hydrophilic active ingredients, needs to release the entrapped species at a controlled-rate so as to maintain a constant dosage range over an extended period of time. At the same time, the matrix forming candidates need to be bio- and environmentally compatible and should be commercially viable. Current limitations in controlled- and extended-release technology involves difficulties in attaining extended temporal control, premature (burst) release of the active agent, manufacturing and materials cost, and unwanted interactions of the matrix-forming materials with biotic components resulting in non-specific side-effects. A large cohort of polymers and biomaterials has been employed as release retarding materials each of which presents a different approach to the matrix concept. For example, poly (hydroxy ethylmethacrylate) (PHEMA), poly(vinyl alcohol) (PVA), poly(vinyl pyrrolidone), polyethylene oxide, sodium alginate [3], polyethylene glycol (PEG) [4,5], hydroxypropyl methylcellulose (HPMC) [6,7,8], and hydroxyethyl cellulose (HEC) have been extensively used which impart control over active release through the formation of insoluble or hydrogel networks [9,10]. Polymers forming insoluble or skeleton matrices, such as biodegradable polyanhydrides [11], polyhydroxyalkanoate [12], polyesters of poly(glycolic acid) (PGA), poly(lactic acid) (PLA), polylactide-*co*-glycolide (PLGA) [10], polyurethanes; or non-degradable poly(dimethylsiloxane) (PDMS), polyethylene vinyl acetate (PVA), and polyvinyl chloride (PVC); poly(vinyl pyrrolidone) or poly(vinyl acetate) are extensively used to generate controlled-release delivery systems for drugs and biologically active macromolecules such as large peptides and proteins. Natural materials, of a hydrophobic and water-insoluble nature, such as lipids, waxes, or fatty acids such as Carnauba wax, beeswax [13], micro crystalline wax [14], candelilla wax [15], ozokerite wax, and paraffin waxes, which are potentially erodible, have also been used as the more economically-viable and environmentally-friendly material candidates to generate controlled-release platforms. Such lipid-based systems have drawn particular attraction due to the advantageous properties they offer in comparison to most synthetic polymers. These properties include an ability to load and release both hydrophilic and hydrophobic compounds, the superior biocompatibility profile of the matrix-forming agents, non-specific interactions with biological components, lower immunogenicity [16,17] and degradation to biocompatible end-products unlike many synthetic polymers [18]. Although lipid-based matrices show a lot of promise in designing extended release formulations, owing to high hydrophobicity, these systems also result in unreliable release kinetics, oftentimes resulting in incomplete or sub-optimal diffusion of the active agent from within the matrix interior. In addition, many of the FDA-approved hydrophobic matrix-formers, such as beeswax, carnauba wax, glyco- and phospholipids, possess complex chemical structures and exhibit significant variability in physical properties depending on the source and method of extraction. Semi-synthetic and synthetic bio-based lipid-like materials are viable options to bypass these limitations of natural lipids and wax based matrix-forming agents, and can offer the benefit of batch-to-batch uniformity, affordability, biodegradability, low toxicity, accessibility, and straightforward synthesis of drug delivery systems [19,20,21]. For example, soybean oil derived fatty acids and synthetically modified products thereof have gained popularity due to facile preparative methods, tunable properties, versatility, low cost, biodegradability, and high bio-based content [22,23]. Epoxidation of triglycerides of soybean oils yields multi-functional lipid-like molecules amenable to application-guided post-synthetic modifications [24]. Oxirane ring opening reaction of epoxidized triglycerides derived from soy has been used for crosslinking modified triglycerides with a variety of acids and alcohols for facile preparation of coatings and film formulations [22,23,25]. Epoxidized sucrose soyate (ESS, Figure 1a), for instance, is a sucrose ester derivative of soybean oil fatty acids, which has been studied for its capability to form cross-linked networks with excellent mechanical properties [25,26]. Soybean oil and its derivatives have also been studied extensively for their applications in polyurethane production, polyesters, and potential application in biomaterials [21,23,27,28]. 

In this study, we report a readily compoundable fabrication process of a hydrophobic matrix system composed of ESS that can be used to fabricate extended release delivery formulations of hydrophilic substances in the form of matrices. To realize this goal, ESS was cross-linked with citric acid (CA) in the presence of the model water-soluble compound, fluorescein sodium salt (Sod-FS), to entrap the latter in a fully bio-based 3D matrix [26]. Sod-FS is a salt of a free basic dye, fluorescein, and has been used in this study as a representative molecule of compounds and drugs which are highly hydrophilic in nature. Citric acid makes a suitable biocompatible cross-linker as it possesses multiple functional groups and capable of fast gelling with epoxides [29,30]. We evaluated the capacity of ESS-derived matrices to sustain the release of hydrophilic Sod-FS, investigated the kinetics of active release as a function of matrix formulations and mechanical properties, and finally evaluated the biocompatibility of the ESS as a matrix-forming material. We envision that such bio-based matrices can provide easy access towards the preparation of controlled-release delivery systems of water soluble active ingredients, intended for pharmaceutical, diagnostic, and theranostic applications.

## 2. Materials and Methods

### 2.1. Materials

Epoxidized sucrose soyate (ESS), with an epoxy equivalent weight of 243.4 g/eq, was synthesized according to a previously reported procedure [26]. Citric acid, polyethylene glycol methyl ether (PEG, *M*_n_ = 750, 2 and 5 kDa) and fluorescein sodium salt were purchased from Sigma-Aldrich (St Louis, MO, USA). Tetrahydrofuran (THF) and dimethyl sulfoxide (DMSO) were acquired from Millipore Sigma (Billerica, MA, USA). Phosphate buffer saline (PBS) tablets (10 mM) were purchased from VWR (Solon, OH, USA), which was used to prepare PBS solution of pH 7.4. All reagents were used as received. L929 mouse fibroblast cell line was purchased from American Type Culture Collection (Manassas, VA, USA). Eagles minimum essential medium (EMEM), Hank’s balanced salt solution (HBSS), fetal bovine serum (FBS), penicillin and streptomycin were acquired from HyClone Laboratories (GE Healthcare Life Sciences, Logan, UT, USA). 3-(4,5-dimethylthiazol-2-yl)-2,5-diphenyltetrazolium bromide (MTT) reagent was purchased from Promega (Madison, WI, USA)

### 2.2. Preparation of ESS-CA Matrix

Soy-derived hydrophobic ESS-CA matrices were prepared by crosslinking ESS with CA following the composition and curing conditions listed in Table 1. First, citric acid was dissolved in deionized water to achieve a 1:6 or 1:8 acid to water molar ratio. In the following step, ESS was added to the CA solution in a glass vial and manually stirred for 2 min followed by high speed mixing in a VWR standard vortex system (VWR, Radnor, PA, USA) until a homogenous mixture was achieved. The mixture was then heated at 80 °C with magnetic stirring for up to 3 h and allowed to cool to room temperature (Figure 1c). For the preparation of Sod-FS loaded matrices, the active agent was dissolved in deionized water prior to adding CA. ESS was then added to the CA-Sod-FS solution to form the matrix in a similar fashion as detailed above. The Sod-FS loading was adjusted to achieve 0.026 wt % (low [Sod-FS]) and 0.065 wt % (high [Sod-FS]) by weight of ESS. For inclusion of release modifiers within the formulation, different molecular weight of PEGs was incorporated at 5 *w*/*v* % aqueous solution prior to adding CA and Sod-FS.

### 2.3. Determination of Loading Content

ESS-CA matrices loaded with Sod-FS were cut into cubes (*v* = 3.41 mm^3^) for each formulation. For estimation of active loading, the cubes were homogenized using a mortar and pestle and incubated in 10 mM phosphate buffer saline (PBS, 10 mL) for 24 h at 37 °C with moderate stirring. The samples were centrifuged at 5000 rpm for 1 h and then filtered through a 0.45 mm micro-filter. The absorbance spectra of the filtered supernatant were analyzed using a Varian Cary 5000 UV–Vis NIR spectrophotometer (Agilent Technologies, Santa Clara, CA, USA) to quantify the Sod-FS concentration. 

### 2.4. Characterization and Measurements

#### 2.4.1. Attenuated Total Reflectance-Infrared (ATR-IR) Spectroscopy

ATR-IR of the components used to fabricate the ESS-CA matrices was performed using a Nicolet 8700 FT-IR with a Smart iTR diamond tip accessory (Thermo Scientific, Grand Island, NY, USA). Spectra representative of the top, bottom, and internal surface of the matrices were collected. To analyze the internal surface, the matrices were sectioned along the transverse plane. 

#### 2.4.2. Gel Permeation Chromatography

A gel permeation chromatograph (GPC) was obtained using a TOSOH EcoSEC HLC-8320GPC system (TOSOH Bioscience, Japan) gel permeation chromatograph calibrated with polystyrene standards. ESS solution was prepared in THF at a concentration of 5 mg/mL.

#### 2.4.3. Viscoelasticity TESTING

Viscoelasticity of the ESS-CA matrices was analyzed using an ARES-G2 rheometer (TA Instruments, New Castle, DE, USA). Matrix samples were cut into 1–2 mm thick discs of 25 mm diameter using a 25 mm hollow steel punch. Samples were analyzed between two parallel plates at 37 °C using a frequency sweep method (0.02–628 rad/s) under a constant 0.5% strain (within the linear viscoelastic region of the samples). The samples were equilibrated for 2 min prior to testing.

#### 2.4.4. Water Contact Angle Measurement

Contact angle of the ESS-CA matrices of different formulations with and without PEGs was measured using FTA 200 dynamic contact angle/surface tension analyzer and FTA 32 video (First Ten Ångstroms, Portsmouth, VA, USA). Measurements were taken from both the top and bottom surfaces of the matrices (*n* = 5). A total of 3 sample replicates were used for each formulation for each surface. 

#### 2.4.5. Content Release Studies

Different formulations of ESS-CA matrices containing Sod-FS (in the forms of cubes of 3.41 mm^3^) were placed in 15 mL of PBS (10 mM, pH 7.4). The samples were incubated at 37 °C with moderate stirring. After every 24 h, 1.5 mL of sample solution was collected and replaced with an equal volume of fresh PBS (10 mM, pH 7.4). The absorbance spectra of the withdrawn sample were analyzed using a Varian Cary 5000 UV–VIS NIR spectrophotometer (Agilent Technologies, Santa Clara, CA, USA). Cumulative percent release was determined using the following (Equation (1)):

(1) $$cumulative\;\%\;release=\;\frac{volume\;withdrawn}{volume\;of\;bath}\;\times \;\%release\;at\;time\;t+\;\Sigma \;\%release\;at\;t-1$$

#### 2.4.6. Scanning Electron Microscopy (SEM)

To analyze the microstructures of ESS-CA matrices, SEM image analysis was performed. Samples were mounted on aluminum mounts using carbon adhesive tabs/tape and then coated with a conductive layer of carbon in a high-vacuum evaporative coater (Cressington 208c, Ted Pella Inc., Redding, CA, USA). Images were obtained with a JEOL JSM-7600F scanning electron microscope (JEOL USA Inc., Peabody, MA, USA) operating at 2 kV. 

#### 2.4.7. Kinetic Analysis of the Release Data of SOD-FS from ESS-Matrices

The dissolution data for Sod-FS from within the ESS-CA matrices of different formulations were fitted to Higuchi equation (Equation (2)):

(2) $$\frac{M}{M_{\alpha }}=kt^{1/2}$$

where $\frac{M}{M_{\alpha }}\;$ indicates fractional release of the linear segment of the release profile, *t* is the time of release, and *k* is a Higuchi constant. As Higuchi equation does not include the swelling and erosion component of diffusion, release data have also been fitted with well-known bi-exponential model (Equation (3)):

(3) $$\frac{M}{M_{\alpha }}=kt^{n}$$

where $\frac{M}{M_{\alpha }}\;$ is the fractional release of linear segment of the release profile, *t* is the release time, *k* is a constant incorporating the properties of the macromolecular polymeric systems and the active ingredient, and *n* is the kinetic constant, which describes the mechanism of molecule transport. For matrices, an *n* value of 0.45 obtained from Equation (3) defines the profile to follow Fickian (Case I) release, 0.45 < *n* < 0.89 describes non-Fickian (Anomalous) release, *n* = 0.89 represents Case II (Zero order) release, and *n* > 0.89 refers to super case II release [31,32]. Fickian (or Case I) diffusion refers to the kinetic mechanism where the polymer relaxation time during molecular release is significantly longer than the characteristic solvent diffusion time [33]. While, in a non-Fickian diffusion mechanism, polymer relaxation time during molecule release and the characteristic solvent diffusion time are equal. Typically, matrix-type devices exhibit Fickian behavior, as it accounts for concentration gradient, diffusion distance, and the degree of swelling [34,35]. Case II diffusion, on the other hand, refers to the dissolution of the polymeric matrix due to the relaxation of the polymer chain, which is independent of the concentration. 

Mean dissolution time (MDT), which is a model independent parameter characterizing active release from a matrix system, was calculated from the Sod-FS dissolution data according to Mockel and Lippold [36] using the following (Equation (4)):

(4) $$MDT=\left(\frac{n}{n+1}\right)\cdot k^{\frac{-1}{n}}$$

### 2.5. Cytotoxicity Studies of ESS

The cytotoxic effect of the molecular form of ESS was tested on L929 mouse fibroblasts. L929 mouse fibroblast cells were maintained in EMEM supplemented with 10% fetal bovine serum (FBS), penicillin (100 U/mL), and streptomycin (100 μg/mL). The cells were incubated for 24 h at 37 °C (5% CO_2_), after which, cells were trypsinized, re-suspended into fresh supplemented EMEM, and seeded into 96-well cell culture treated plates at an inoculum density of 50,000 cells/mL. The plates were incubated for 24 h at 37 °C (5% CO_2_) and EMEM was carefully removed using a multichannel pipette and attached cells were rinsed once with HBSS. Pure ESS solutions prepared in HBSS (0.01–100 µM) were transferred in triplicate to the rinsed, pre-cultured cells and incubated for 24 and 72 h at 37 °C (5% CO_2_). Bright field microscopy images were acquired on inverted microscope prior to removal of ESS containing media for cell viability assessments. After removal of spent media, cells were rinsed 3 times with HBSS. A 0.5 g/L solution of MTT in HBSS (0.2 mL) was added to each well and incubated for 4 h at 37 °C. The MTT solution was removed from each well and 0.15 mL of DMSO was added. The plates were placed on an orbital shaker (150 rpm; ambient temperature) for 15 min to solubilize the MTT dye. Absorbance values were measured at 570 nm using a multi-well plate spectrophotometer, Tecan Safire 2 microplate reader (Tecan, Switzerland).

## 3. Results

### 3.1. Fabrication of the Matrices

Epoxidized sucrose esters of fatty acids have been reported as platform materials for bio-based epoxy resin technology due to the presence of a compact molecular structure, multivalent epoxy group functionality, and high density [25,37]. As represented in Figure 1a, ESS, which is composed of soybean oil fatty acid esters, has been shown to form excellent cross-linked materials where the rigid core of sucrose yields a desirable mechanical property, and the presence of multiple epoxy functionalities render the materials amenable to cross-linking induced by multi-functional carboxylic acid or anhydrides [26]. With a fairly monodispersed molecular weight of 3000 g/mol (Figure 1b), ESS has a high content of ester linkages within its structure, and curing with bio-based or natural carboxylic acids that cross-link the 3D matrix provides a mechanism for biodegradation of the scaffold and the controlled release of any incorporated active molecules. We have utilized the CA mediated cross-linking methodology of ESS and developed a facile procedure to homogeneously disperse the hydrophilic active agent within the cross-linked matrix of ESS (Figure 1c). Ma et al. [26] reported the cross-linking efficiency of CA with ESS for the first time, and found out that, depending on the proton concentration, a cross-linking time of at least 20 min is required to cross-link all the epoxy groups of the sucrose ester by the acid at an equivalent molar ratio, as indicated by the measurement of gel time of the mixture. In our case, ESS-CA matrices were cured at 80 °C for up to 3 h, after which the mixture formed solid, 3D, translucent thermoset matrices. The organoleptic properties of which are usually varied with the incorporation of the active ingredient and with different release modifiers (Figure 1c). One of the reasons for selecting citric acid to cross-link ESS epoxy groups was the moderate p*K*_a_ value of the acid, so that the pH of the local environment post-degradation is not altered significantly. Acidification of local pH upon degradation is one of the major problems with matrices prepared from PLGA or PLA, due to the production of lactic acid on hydrolysis. ATR-IR spectroscopy of a representative sample of the matrices (Figure 1d) demonstrates the presence of strong signals at 1750 cm^−1^, indicating the presence of ester bonds of ESS, which is a critical structural component for ensuring biodegradation and the controlled release mechanism of the matrices.

### 3.2. Surface, Microstrutural and Mechanical Property Analysis of ESS-CA Matrices

**Figure 2:**
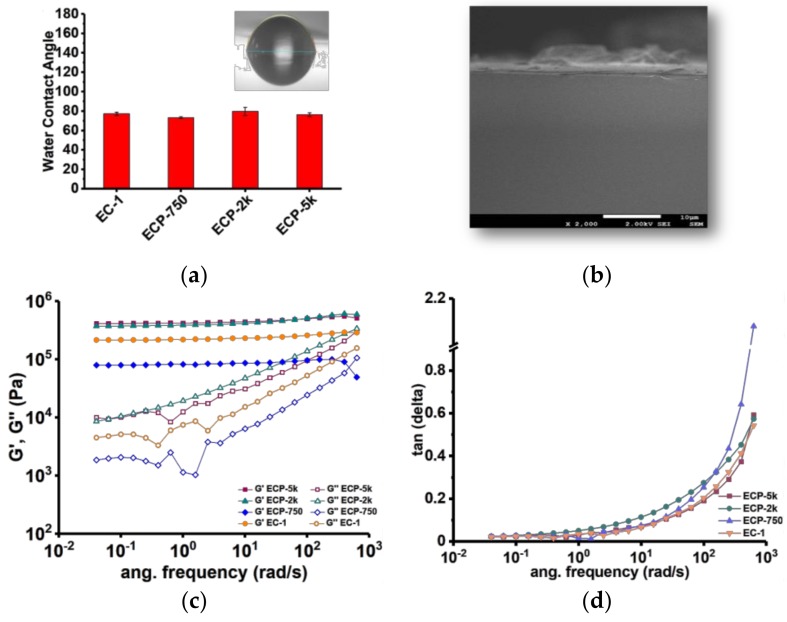
(**a**) Water contact angle on top surfaces of the ESS-Citric Acid matrices (insert: image of typical water droplet on surface of the ESS-citric acid matrix); (**b**) Scanning electron microscopy (SEM) image of ESS-citric acid matrix loaded with Sod-FS; (**c**) *G*’ modulus curve of control and PEG modified ESS citric acid matrices; (**d**) *G*” modulus curve of control and PEG modified ESS citric matrices.

Contact angle measurement of water droplets on ESS-CA matrices indicated that the matrices have a highly hydrophobic surface. Irrespective of formulation, contact angle of matrix surfaces which were in contact with air were found to be within the range of 75–80°. Representative drop shapes of water on ESS-CA matrices are illustrated in Figure 2a (insert) along with the calculated water contact angle as a function of formulations. Inclusion of PEG, at least in the tested weight ratio, did not change the surface hydrophobicity of matrices. Such surface hydrophobicity is significant for a matrix system, since an apolar surface will help to suppress the burst release of the active ingredient located on matrix surfaces when immersed in aqueous environment, thereby favoring more controlled release of the active substance.

The internal microstructure of ESS-CA matrices was determined by capturing the SEM image of lateral cross section of matrices prepared with different formulations (Figure 2b). The SEM micrographs revealed that ESS-CA matrices alone possess a non-porous and uniform microstructure, indicating the formation of a monolithic matrix. No gross microstructural changes or defects were evident even after the addition of PEGs or Sod-FS within the matrix. SEM experiments indicated that ESS-CA matrices possess a homogenous microstructure, where the incorporated active agent is evenly distributed. Such monophasic distribution of the latter is critical for a highly controlled and monotonic release rate of the incorporated species from a macromolecular scaffold. 

We have also analyzed the viscoelastic properties of different formulations of EC matrices and evaluated their loss and storage modulus as a function of the release-modifiers (i.e., PEGs of different molecular weights) being incorporated within the systems. We observed that the inclusion of high molecular weight PEGs (at 5% *w*/*w* of total formulation) increases both the loss and storage moduli of the matrices (Figure 2c), compared to those prepared without PEG (EC-1). Inclusion of low molecular weight PEG (i.e., 750 g/mol), decreased both the loss and storage moduli. More crystalline structures and higher strength/modulus of the high molecular weight PEGs are most likely to be the contributing factors for such observations, indicating that the matrices were more visco-elastic in consistency than rubbery (Figure 2d).

### 3.3. Content Release Studies

**Figure 3:**
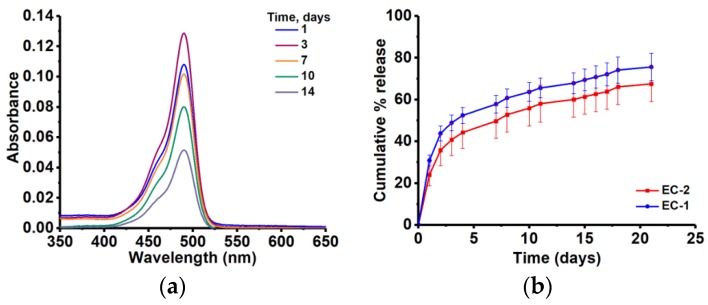

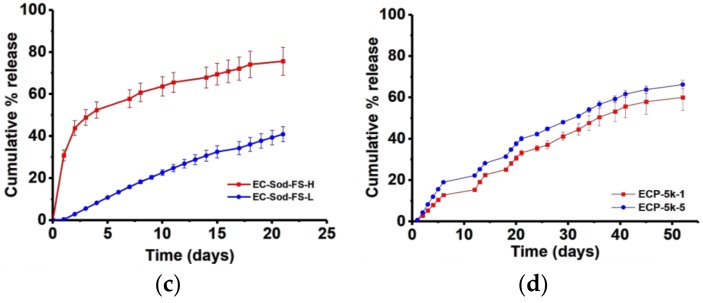
(**a**) Example of absorption spectra of Sod-FS released from a typical ESS-citric acid matrix; Effect of (**b**) acid concentration in formulation; (**c**) loading concentration (**d**) amount of PEG in a modified matrix on the release of Sod-FS.

Active release from non-porous lipid matrices occurs through diffusion of molecules from the polymer matrix and in some cases through erosion of the polymer matrix. The ESS-CA based matrices designed in this study were intended for use as a molecular delivery platform, particularly for controlling the release of a small, hydrophilic active molecules for an extended period of time. In clinical settings, such matrices are important for designing delivery systems for contraceptive agents, growth factors, enzymes, analgesics, and orthopedic medications, and antibacterial compounds. We have used Sod-FS as such a model small molecular agent for our study. A tightly controlled diffusion of Sod-FS, spanning over a period of more than 20 days, was observed from all the ESS-CA derived lipid matrices (Figure 3). First we tested if the curing process alters the chemical functionality of the model compound. Figure 3a shows the UV–Vis spectral profiles of Sod-FS indicating the compatibility of the agent with matrix forming materials and processes. The signature absorption signal of Sod-FS is observed even after the 14-day release. Cross-linked ESS matrices without PEGs (EC-1 or 2) did not show any signs of scaffold disintegration within this time period (Figure 3b). This finding is in accordance with the work carried out by Dakkuri et al. [38] with wax type matrices.

Crosslinked ESS matrices with no PEGs are extremely hydrophobic in nature with lower wettability. Complete release of Sod-FS from an EC-1 or 2 matrix system was not possible since a certain fraction of the active molecule is always coated with impermeable hydrophobic film. In addition, it has been reported earlier that in the absence of additives, active release is oftentimes prolonged and non-linear from hydrophobic matrix systems [39]. Since our formulations contain no channeling agents, formation of pores and cracks did not occur to facilitate Sod-FS release. As a result, the water-impervious hydrophobic EC matrices showed sustained Sod-FS release for almost over a month. Figure 3c shows the effect of acid concentration on the release profile of Sod-FS from EC matrix (EC-1 vs EC-2). Formulations cross-linked with higher acid concentration (EC-1), released up to 70% of the FSS within 21 days, while formulations cross-linked with lower acid concentration (EC-2) released 10% less Sod-FS cumulatively within the same time period. The steep increase of Sod-FS release for the first 5 days could be attributed to the Sod-FS molecules accumulated at or near the surfaces of ESS-CA matrices. 

A set of factors such as loading content of the active ingredient, amount, and molecular weight of PEGs that have been included as release modifiers were found to influence the release of Sod-FS from the ESS matrix. Figure 3c shows the amount of active loading on release rate. High drug loading led to a burst release of Sod-FS from matrices and close to 30% of the entrapped compound was released within the first 48 hours from this formulation (EC-Sod-FS-H). On the other hand, reducing drug loading helped formulation EC-Sod-FS-L to achieve almost a zero-order release profile liberating approximately 40% of the incorporated amount of Sod-FS over 20 days. Inclusion of PEG resulted in a more uniform release of Sod-FS over extended period of time (>60 days) and suppressed burst release (Figure 3d). Extent of Sod-FS release was also found to depend on the amount of PEG included in the formulations. Inclusion of 5% *w*/*w* of PEG (5kDa) per matrix formulation resulted in a higher amount of Sod-FS release compared to that containing 1% *w*/*w* of PEG of the similar molecular weight. The rate of Sod-FS release, if not extent, was found to depend on the molecular weight of PEGs and amount (wt %) of PEG incorporated as illustrated in Figure 3d, most likely due to the differences of aqueous solubility of PEGs of different molecular weight.

### 3.4. Release Kinetics

**Figure 4:**
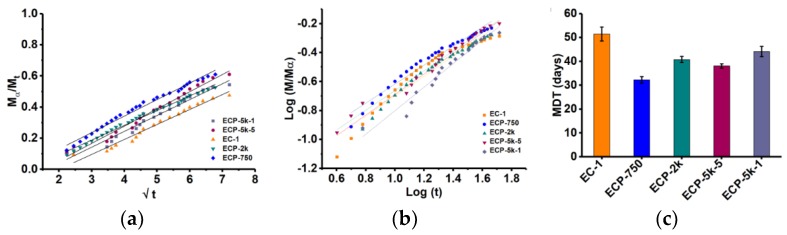
Kinetic analysis of active release by (**a**) Higuchi mode; (**b**) Biexponential model; and (**c**) Mean Dissolution Time of Sod-FS (in days) from different ESS-derived matrices.

Mathematical models were used to determine the mechanism of solute transport from the matrices, such as Higuchi (Equation (2)) and biexponential equation (Equation (3)). Linearity of Higuchi-type release kinetics (fractional release vs. square root of time) is an indicator of diffusion of the active compound occurring from a typical matrix-based system, where the active ingredient is homogenously dispersed within a consolidated polymeric phase. As evident from Figure 4a, incorporation of high molecular weight PEGs, that is, 2 or 5 kDa, caused the release profile to follow Higuchi kinetics compared to those containing low molecular weight PEGs, or without PEGs. Figure 4b describes the fitting of the linear fraction of Sod-FS release in the biexponential model (Equation (3)). 

Values for release exponent (*n*), kinetic rate constant (*k*) obtained using Equation (3) (*R*^2^ > 0.96) is reported in Table 2. The n values obtained using the biexponential model also showed that matrices composed of only ESS and CA (i.e., EC-1) exhibited non-Fickian (anamolous) diffusion mechanism. Earlier work with wax matrix systems also revealed that pure hydrophobic matrix-forming agents oftentimes followed non-Fickian type of release behavior irrespective of physico-chemical nature of the drug, making it difficult to draw any clear inference regarding the kinetics of active release from such matrices [6,40]. In our cases, the mechanism of Sod-FS release could be attributed to the rate of fluid entry through the cracks and pores of the matrices which initiates the diffusion of Sod-FS out of the matrix. However, due to high hydrophobicity of the matrix surface, such fluid entry through the matrices, was insignificant [41,42], resulting in significant deviation of *n* value from Fickian or diffusion controlled mechanisms for EC-1 formulations. The effect of PEGs on release kinetics of Sod-FS from the hydrophobic matrices is also evident when the release rate of the later is compared among different formulations. Incorporation of PEG within the matrix decreases the *n* value towards Fickian (Case I diffusion-controlled) behavior (Table 2). The presence of PEG in the formulations increases the wettability of the matrix surfaces, thereby contributing to diffusional component of release kinetics for a highly water soluble active compound such as Sod-FS. 

Using the intercept value (*k*) obtained from the biexponential model, mean dissolution time (MDT) was calculated from Equation (4). MDT value is used to characterize the release rate of the active ingredient from a delivery system and the retarding efficacy of the polymer. A higher value of MDT indicates an efficient active retaining ability of the polymer and vice-versa. As represented in Figure 4b, the highest MDT value was associated with EC-1 matrices, which does not contain any PEG (51 days), and the lowest MDT value of 30 days was found with ECP-750 matrices containing PEG of an Mn value of 750 g/mol. Rapid diffusion of small molecular weight PEG out of the matrix skeleton might be an attributable factor for such an observation. Inclusion of PEGs of higher molecular weight, that is, 2 or 5 kDa, increases the MDT value by approximately 10 days, most likely due to molecular entanglement and swelling associated with high molecular weight PEGs.

### 3.5. Cytotoxicity Assessment

**Figure 5:**
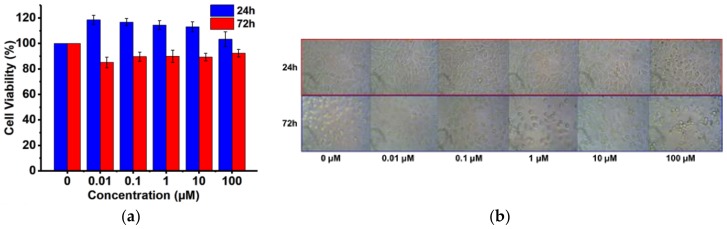
(**a**) Cytotoxicity assessment of ESS in Mouse L929 fibroblast cells; and (**b**) Morphology of mouse L929 fibroblasts after 24 and 72 h treatment with increasing concentration of ESS.

Upon evaluation of the materials and kinetic properties of the ESS matrices, we determined the biocompatibility profile of the major matrix forming agent, ESS, using L929 mouse fibroblast cells. Cells were treated with an increasing concentration of ESS for 24–72 h at 37 °C, and the cell viability was quantified at the end of the treatments using an MTT colorimetric assay. Although epoxy functional groups were present in the ESS molecule, to our surprise, we did not observe any severe cytotoxic effects when compared to non-treated cells after 24 or 72 h of exposure. As a macromolecular construct, ESS by itself was found to be non-toxic (>85% cell viability) at concentrations as high as 100 μM (Figure 5a). We have also evaluated the morphology of L929 fibroblast cells with bright field microscopy (Figure 5b). When compared to control, no severe phenotypic or morphological changes were evident in mouse L929 cells, indicating excellent biocompatibility of the matrix forming materials towards mammalian cell lines. Biocompatibility of ESS is most likely due to the very low cytotoxic potential of the molecular components of ESS, that is, soybean oil fatty acid and sugar, which are connected through ester bonds. Hence, cellular and metabolic degradation of ESS most likely generate non-toxic end products. Soy-based products, that is, hydrogenated soybean oil (NTIS accession number, PB266280) and soy protein isolate (NTIS accession number, PB300717) have long been classified as GRAS (Generally Regarded as Safe by FDA) rendering the material suitable for human use (accessdata.fda.gov).

## 4. Conclusions

We have evaluated a new bio-based material, ESS, for fabrication of controlled- and extended release matrices for water-soluble small molecules. We observed that ESS provided dual benefits of having cross-linkable epoxy groups for imparting structural rigidity and hydrolysable ester bonds suitable for biodegradation within the same molecule. Hence, it was possible to readily generate a freestanding, biocompatible, and biodegradable 3D matrix that can entrap and extend the release of a hydrophilic model substance for over a month. The mechanical and physico-chemical properties of these new matrix-forming materials have been investigated and are correlated with their molecular loading and release capacity. ESS behaves like a wax-type matrix forming material as evident from release kinetic analysis. By incorporation of release modifiers such as PEGs, it was possible to attain the diffusion-controlled release profile of the model active compound from these matrices without burst release. We envision that such platform technology will find a multitude of applications, such as to design point-of-care extended release drug and cosmetic delivery systems, and to prepare biological scaffolds. We are currently working on rendering the ESS platform stimuli-responsive so as to generate smart materials for medical and diagnostic applications.

## Figures and Tables

**Table 1:** Compositions and Curing Conditions for ESS-Citric Acid Cross-linked Matrices.

Sample	Molar Ratio					*T* °C/Time	Matrix Type
	ESS	Citric Acid	Di-H_2_O	PEG	Sod-FS		
EC-1	1	0.98	6			80/2 h	High [H^+^]
EC-2	1	0.98	8			80/2 h	Low [H^+^]
ECP-750	1	0.98	6	0.007		80/3 h	PEGylated Matrices
ECP-2k	1	0.98	6	0.007		80/3 h	
ECP-5k-5	1	0.98	6	0.007		80/3 h	
ECP-5k-1	1	0.98	6	0.003		80/2.5 h	
EC-Sod-FS-L	1	0.98	6		0.002	80/2.5 h	Low [Sod-FS]
EC-Sod-FS-H	1	0.98	6		0.005	80/2.5 h	High [Sod-FS]

* E = ESS, C = Citric Acid, P = PEG methyl ether, Sod-FS = fluorescein sodium salt.

**Table 2:** Release kinetics values of the release exponent (*n*), kinetic constant (*k*) and correlation coefficient (*r*^2^).

Sample	*n*	*k*	*r*^2^
EC-1	0.8678 ± 0.0527	0.0171 ± 0.0034	0.9662 ± 0.0038
ECP-750	0.7097 ± 0.0118	0.0456 ± 0.0034	0.9639 ± 0.0096
ECP-2K	0.7238 ± 0.0206	0.0366 ± 0.0037	0.9725 ± 0.0044
ECP-5K-5	0.6802 ± 0.0122	0.0455 ± 0.0018	0.9805 ± 0.0026
ECP-5K-1	0.7972 ± 0.0474	0.0258 ± 0.0041	0.9725 ± 0.0023
